# The evolving panorama of vascular access in the 21st century

**DOI:** 10.3389/fneph.2022.917265

**Published:** 2022-10-26

**Authors:** Nilda Roxana Neyra, Shoaib Wazir

**Affiliations:** Arizona Kidney Disease and Hypertension Center (AKDHC), Phoenix, AZ, United States

**Keywords:** vascular access, arteriovenous grafts, arteriovenous (AV) fistula, hemodialysis catheters, angioplasty, infection

## Abstract

There are three major types of hemodialysis vascular access: hemodialysis catheters, arteriovenous grafts, and arteriovenous fistulas. Arteriovenous fistulas provide the best access due to their reliability and long-term patency. They are recommended by the current Kidney Disease Outcomes Quality Initiatives (K-DOQI) guidelines; however, not all patients benefit from arteriovenous fistulas due to poor maturation or a lack of adequate vasculature. Currently, hemodialysis is initiated *via* catheters in the majority of patients. Catheters are associated with high morbidity and mortality due to infection, lower quality of dialysis, and the development of central vein stenosis. The varied responses of patients to the different access types exemplify the need to choose the “right access for the right patient” based on scores that can predict death risk and progression of chronic kidney disease. Additionally, vascular access, often referred to as the “Achilles’ heel” of hemodialysis patients, represents a significant percentage of the Medicare budget that continues to increase yearly. The purpose of this paper is to review the current literature on the management of vascular access complications and infection treatment and prevention. The paper also explores emerging research regarding the devices and methods to improve access outcomes such as early cannulation arteriovenous grafts, endovascular arteriovenous fistula creation, and regenerative grafts with resorbable scaffolds, among others. The data were collected through literature searches *via* PubMed, Athens and web search engines.

## Introduction

Hemodialysis is a rather recent invention. In 1924, Haas was the first to perform hemodialysis in a human. Since then, hemodialysis has undergone several improvements. First, the Scribner shunt was created in 1960 and permitted repeated dialysis sessions without the risk of thrombosis. Then, the development of the native arteriovenous fistula (AVF) by Cimino-Brescia in 1966 set the path for chronic hemodialysis. Today, hemodialysis benefits 70 to 90% of renal replacement therapy patients (1, 2).

Chronic hemodialysis has contributed to the long-term survival of people with end-stage kidney disease (ESKD), as well as patients who have recovered from acute kidney injury with dialytic support. Furthermore, patients with multiple comorbidities, such as heart failure and other end-organ damage, can survive due to support from hemodialysis. For these reasons, the number of patients requiring hemodialysis has increased significantly worldwide. Currently, four million people require hemodialysis worlwide, with the highest incidence of treated patients in Mexico, Taiwan, Hungary, and the United States (U.S.). From 2000 to 2018, the number of hemodialysis patients almost doubled in the U.S. (252,212 to 485,052 patients) (2).

Achieving functioning vascular access in a dialysis patient is a challenging endeavor. To provide adequate hemodialysis, proper vascular access that can achieve more than 300 ml/min of blood flow is needed. There are three main types of hemodialysis accesses, including tunneled and nontunneled catheters, arteriovenous fistulas (AVFs), and polytetrafluoroethylene (PTFE) arteriovenous grafts (AVGs). Among the three available modalities, AVFs are superior because of their excellent reliability, best patency, low rate of infection and thrombosis, and association with reduced mortality (1). Although AVFs have these benefits, only 60% mature to the point of cannulation, highlighting the prevalence of vascular access dysfunction, which is a significant cause of morbidity and mortality in the ESKD patient population (3). Additionally, this dysfunction requires frequent interventions, which make the care of the vascular access costly, and accounts for a significant percentage of the dollars spent annually in the care of ESKD patients (2).

Currently, significant research is being conducted to understand the biology of the vascular access circuit, which is composed of the inflow artery, the outflow vein, and the conduit that directly connects the two. An important concept is the consideration that the vascular access begins at the heart and returns to the heart, and how they are closely interconnected. Fistulas can fail due to significant heart disease and high flow fistulas can induce heart failure as well. Multiple devices and techniques have been developed to improve the maturation, patency and longevity of the different vascular accesses. However, an ideal vascular access has not yet been developed.

Important initiatives have been created to optimize vascular access management. In 2003, the Centers for Medicare and Medicaid (CMS) and the ESKD networks collaborated to implement a National Vascular Access Improvement Initiative called the Fistula First Initiative (FFI). FFI aimed to encourage practitioners to perform early referrals and placement of arteriovenous fistulas in preparation for dialysis (3). In the U.S., the Centers for Disease Control and Prevention (CDC) created the National Healthcare Safety Network (NHSN), which is a national tracking system for infections in patients on dialysis that relies on self-reporting. The development of the NHSN has led to increased tracking of hospitalization events coded as bacteremia-sepsis, allowing for a greater transparency regarding the infection prevalence. The CMS evaluates the option of tracking beyond self-reporting and creating a standardized system to report catheter-related bacteremia (CRB) as event/1000 catheter days. This method is recognized as the most informative way to monitor infections (4). In 2016, the CDC awarded a grant to the American Society of Nephrology to develop a program called the Nephrologists Transforming Dialysis Safety (NTDS). This program aims to encourage nephrologists and dialysis medical directors to achieve a goal of zero infections in hemodialysis facilities. The NTDS program hopes to achieve this goal by improving the systems, implementing collaboration to minimize risk, and creating a culture in the dialysis facility in which patients and staff are encouraged to report safety challenges without fear of reprisal. The NTDS program has a core curriculum that includes infection prevention measures and promotes education *via* webinars (3, 5).

## Arteriovenous fistula

**Figure 1:**
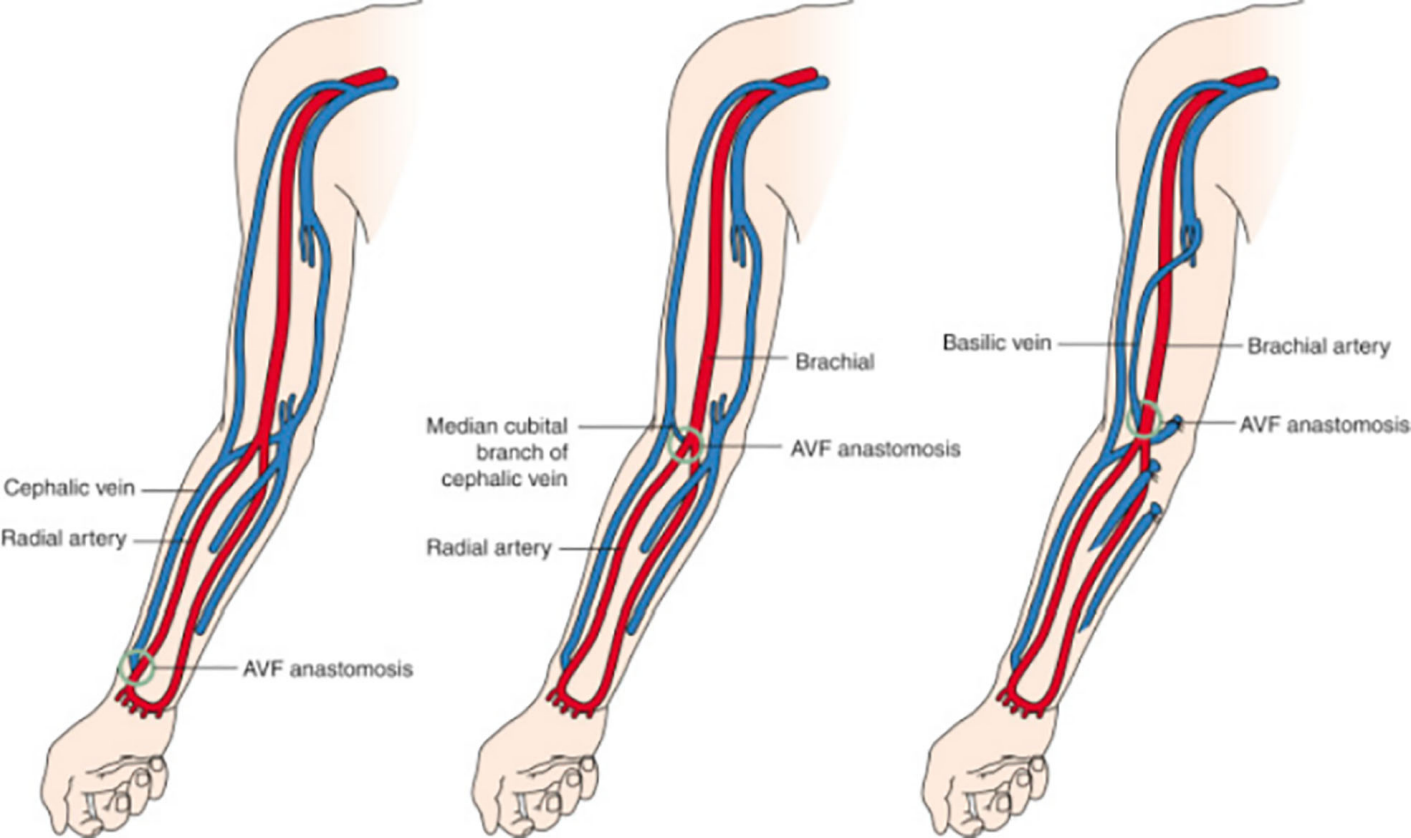
Common types of arteriovenous fistulas. *Left*, Radiocephalic fistula. *Middle*, Brachiocephalic fistula. *Right*, Brachiobasilic transposition. Radiology key.com/arteriovenous fistulas (Redrawn from Allon and Robbin (8); Figures 5-7; used with permission).

As previously mentioned, AVFs are recommended for ESKD patients on hemodialysis due to their many benefits, but they pose challenges. The preferred vessels for AVF creation are the cephalic and basilic veins, which are medium-sized veins of the superficial system of the upper extremities. The most common AVFs are radiocephalic, brachiocephalic, and brachiobasilic transposition. The distal AVF location is the radio-cephalic in the wrist, commonly referred to as the Cimino fistula based on its original creation by Cimino-Brescia in 1966. It is the preferred access due to its association with better patency rates, low complication rates, accessibility and acceptance from patients (1, 4). This statement has been challenged by new reports such as the one by Farrington et al. (6), which showed that upper arm accesses mature better and require less assisted maturation interventions. However, this choice may also trigger other complications such as steal syndrome, high output failure and more frequent aneurysm formation (7) (**Figure 1**).

Unfortunately, only 60% of AVFs are functional at the 12 month mark. Several studies have shown that the patency rates are linked to a range of variables, including older age, presence of diabetes, race, body mass index, smoking, total cholesterol, peripheral vascular disease, female sex and, in some studies, cardiovascular disease. Individual variations explained by genetic susceptibility are associated with vessel characteristics that have poor functional outcomes of AVF. Surgical technique, and expertise of the operator also play a role in successful AVF maturation (7, 9). Although not completely understood, fistula maturation is a dynamic process in which vascular remodeling is facilitated by the release of nitrous oxide and the breakdown of elastin to allow enlargement of the draining vein. When a fistula matures, blood flow through the anastomosing artery can increase up to tenfold; the vein wall thickens and increases in diameter to allow regular cannulation. For maturation, veins prefer nonpulsatile blood flow—AVFs that mature successfully are typically characterized by a soft pulse. Valves in the vein and sites of prior vein injuries, such as intravenous punctures or catheters, may impede outward remodeling and can be sites of stenosis (10). An AVF can undergo maturation failure due to a lack of arterial and venous dilatation and accelerated venous neointimal hyperplasia (11).

The histology of neointimal hyperplasia is characterized by an abundance of contractile, smooth muscle cells, myofibroblasts, and macrophages, which eventually narrow the venous outflow, leading to stenosis and a reduction in blood flow or, in many cases, thrombosis. The proposed mechanisms for neointimal hyperplasia include inflammation, uremia, hypoxia, shear stress, thrombosis, and others. These mechanisms work together through linked cytokine cascades and possibly epigenetic changes that induce negative remodeling, leading to fistula failure (9).

**Figure 2:**
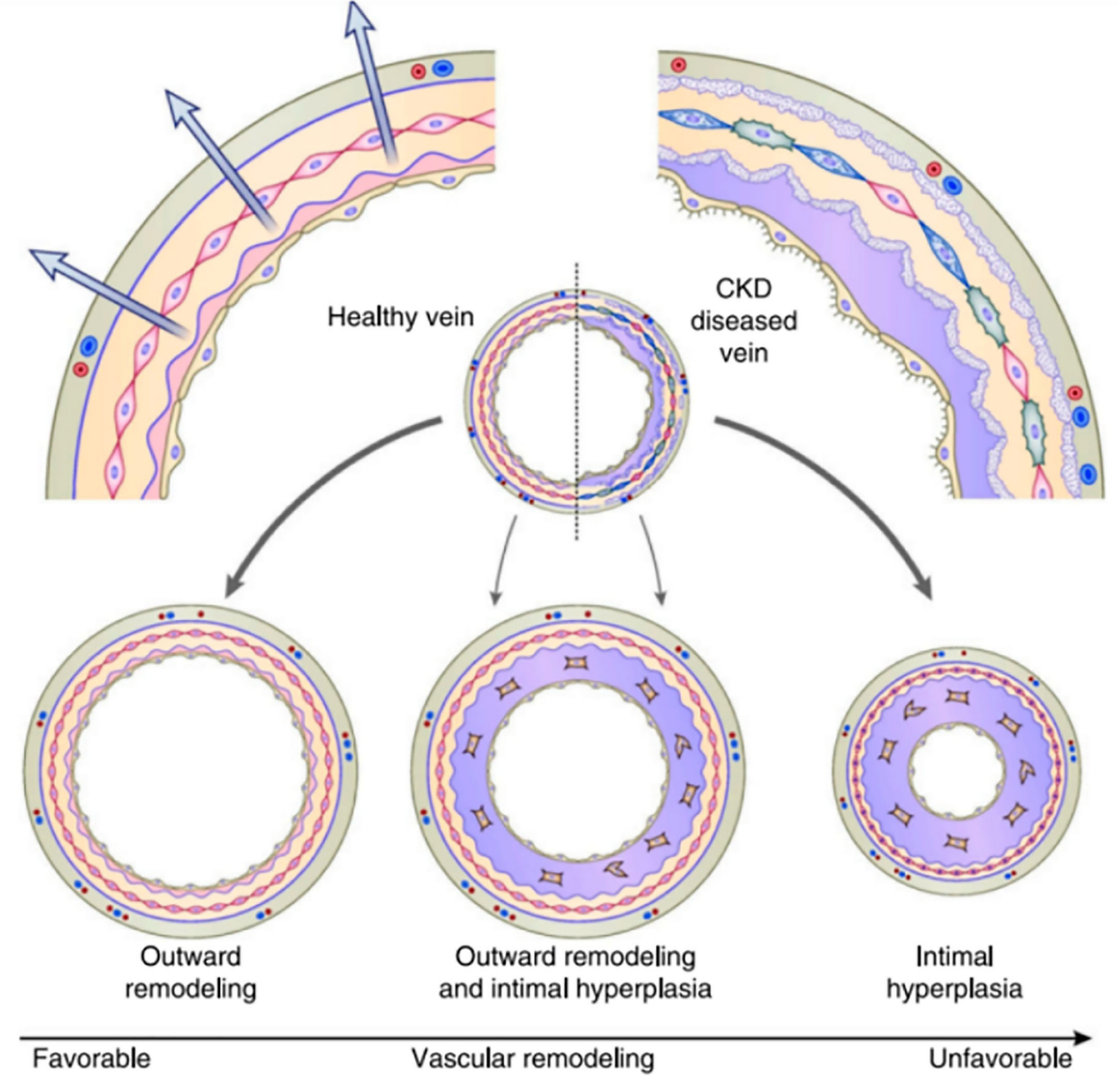
AVF maturation reflects the balance between inward remodeling (intimal hyperplasia) and outward remodeling (vasodilation) Allon (13).

In the hemodialysis fistula maturation study, 602 patients were observed prospectively through AVF creation and maturation. This study examined the association of the preexisting intimal hyperplasia in vein samples obtained at the time of fistula creation and the postoperative AVF venous stenosis detected by serial ultrasounds. The authors did not find a significant association between the incidence of preexisting intimal hyperplasia and the presence of AVF venous stenosis on postoperative ultrasounds (12). These findings have questioned the role of neointimal hyperplasia in fistula maturation failure. This same study showed that preexisting arterial reactivity positively correlated with the 6-week AVF diameter and blood flow. This observation suggests that the ability of the artery to dilate after AVF creation is an important determinant of AVF maturation. AVF maturation depends on the relative balance between neointimal hyperplasia (inward remodeling) and sustained vasodilatation (outward remodeling). AVF maturation failure would occur primarily in the subset of patients with both aggressive neointimal hyperplasia and impaired vasodilation (13) (**Figure 2**).

In 2003, the Fistula First Breakthrough Initiative (FFBI) had a substantial positive impact on improving vascular access outcomes in the U.S. In 1998, the prevalence of AVF use in the U.S. was 26%, and in December 2015, it had increased to 63% (14). This increase improved the vascular access outcomes; however, many fistulas (28 to 53%) fail to adequately mature to support dialysis therapy. The USRDS shows that between June 2014 and May 2016, 39% of the AVFs that were created had failed to mature. For those that mature sufficiently, the median time to first use was 108 days (11). These findings show that fistulas can lead to additional morbidity and may not be appropriate for all patients. Other options, which will be later discussed, are worth considering in certain cases (4).

AVF maturation usually occurs in 4 to 6 weeks. Physical examination and ultrasound provide information concerning the AVF adequacy and the possible causes of nonmaturation that require intervention (15). The National Kidney Foundation’s Kidney Disease Outcomes Quality Initiative (KDOQI) clinical practice guidelines for vascular access recommend following the “rule of sixes,” which states that a mature fistula should achieve a blood flow of at least 600 ml/min, a diameter of at least 6 mm, and a depth of 6 mm or less from the surface of the skin (4). These standards, although practical, do not consider the individual needs of each patient, as some fistulas can achieve adequate dialysis with lower flows (10).

AVFs are associated with three major complications, including aneurysm formation, stenosis and/or thrombosis and arterial steal syndrome, that will be discussed in the next section.

1) Aneurysm formation occurs from repeated cannulation that thins the wall of the native vessel and usually requires surgical repair. If left untreated, an aneurysm can rupture, leading to the risk of life-threatening hemorrhage and possible loss of access (16).

2) Stenosis/thrombosis is reported in up to 60% of functional AVFs (15). Percutaneous transluminal angioplasty is the standard, first-line treatment for venous stenosis (17). However, endovascular interventions may be associated with accelerated neointimal hyperplasia, faster progression of stenotic lesions, and therefore repeated interventions. In recent systematic reviews and meta-analyses, approximately 50% of the patients treated with angioplasty undergo repeat intervention within six months. There is a need to further understand which lesions need to be treated to improve access function without compromising long-term patency. For some authors, this may be a marker of poor vasculature and may justify AVG placement (15). Indications such as occlusions, frequent restenosis, and balloon angioplasty-induced rupture of the vein have resulted in the development of stents to supplement balloon angioplasty and maintain patency of the access to remain functional (9). Cephalic arch stenosis is common among ESKD patients with brachiocephalic AVFs. Physical factors such as valves in the cephalic arch and the course of the cephalic arch through the deltopectoral groove may constrict the blood vessel and limit venous return into the cephalic vein. Dynamic fluid changes, neointimal hyperplasia, and hypertrophic remodeling also contribute. Cephalic arch stenosis is challenging to treat and responds poorly to angioplasty alone, with a primary patency rate of 42% at six months. This is further complicated by higher rupture rates. A recent systematic review and meta-analysis showed the superiority of stent grafts over other endovascular treatment modalities, including angioplasty, bare-metal stents, and drug-eluting stents, in treating cephalic arch stenosis (18). In a retrospective study by Miller and Friedman (19), analysis of patients who underwent flow reduction using the MILLER banding procedure showed a clinically significant reduction in interventions at the cephalic arch. This procedure could be a viable and inexpensive option for patients with high-flow fistulas and cephalic arch stenosis.

Another potential option for the treatment of stenosis is the drug-coated balloon (DCB). Paclitaxel is a commonly used chemotherapy agent for drug-coated balloons. It prevents smooth muscle cell proliferation and thus decreases the risk of restenosis following angioplasty. Kennedy et al. (20) performed a meta-analysis of 12 randomized controlled trials and found drug-coated balloon-paclitaxel-based angioplasty to have improved patency compared to plain balloon angioplasty in maintaining the AVF target lesion patency at 3, 6, and 12 months. The pooled patency at six months was 73.7% for drug-coated balloons versus 55.2% for balloon angioplasty. This analysis had insufficient quality evidence, with significant heterogeneity and imprecision among studies (20). In 2018, a meta-analysis of randomized control trials on femoral peripheral vascular disease was published by Katsanos et al. (21). It raised the concern that paclitaxel-coated balloons were associated with an increased mortality rate, but the exact mechanism remains unknown (21). A recent systematic review and meta-analysis by Dinh et al. (22) compared drug-coated balloons versus angioplasties in dialysis access intervention. It demonstrated no significant difference in all-cause mortality (22). Sirolimus, another antiproliferative agent, is also used in drug-coated balloons. Tan et al. (23) performed sirolimus drug-coated balloon angioplasty in 20 patients with thrombosed upper limb AVG. The primary circuit patency rates at 3 and 6 months were 76% and 65%, respectively, while the assisted-primary circuit patency rates at 3 and 6 months were 82% and 65%, respectively. The 3- and 6-month secondary circuit patency rates were 88% and 76%, respectively. Using Kaplan–Meier analyses, the estimated mean primary, assisted-primary, and secondary patencies were 285 days (95% confidence interval (CI) = 194-376 days), 319 days (95% CI = 221-416 days), and 409 days (95% CI = 333-485 days), respectively. No adverse events directly related to sirolimus DCB use were observed (23). Considering that these data do not demonstrate that DCBs provide a substantial benefit, they are costly, and possibly related to poor outcomes, their use on a regular basis is not justified. Further randomized, large-scale controlled trials are needed to establish the value of drug-coated balloons in vascular access stenosis.

The European Renal Best Practice and European Society of Vascular Surgery recommends the use of far infrared therapy (FIR) A novel noninvasive approach, for AVF non maturation and stenosis. The use of FIR is based on the concept that stenosis is caused by endothelial dysfunction, inflammation and smooth muscle cell proliferation that leads to intimal hyperplasia. Nitric oxide, Heme-Oxygenase, TNF-alpha and MCP-1 are considered to be important in preventing this process. FIR is an electromagnetic radiation therapy (heat therapy) that is applied directly on the skin above the AVF. This therapy has shown thermal and nonthermal effects; thermal effects that produce vasodilatation and angiogenesis and non-thermal effects that possibly inhibit vascular endothelial inflammation *via* stimulation of vasodilating factors such as Heme-oxygenase and nitric oxide production (24). Wan et al. (25) recently performed a meta-analysis of 21 studies and found that FIR therapy can reduce AVFs occlusion and needling pain level, while significantly improving the level of vascular access blood flow, AVF diameter and AVF primary patency. An ongoing randomized, controlled clinical trial is being performed on incident AVFs and existing AVFs for which patients will receive FIR three times per week for 1 year. The primary outcome for incident fistulas will be the maturation time, and the number of interventions compared to controls for the prevalent fistulas. The researchers in this trial will explore this potentially promising treatment modality that could improve AVF maturation and survival (24).

3) Arterial steal with varying degrees of symptoms and will be discussed later.

## Endovascular arteriovenous fistula

EAVF creation offers a new minimally invasive method for fistula creation. WavelinQ and Ellipsys are the two devices utilized in creating endovascular fistulas. EAVFs use the radial artery and radial vein, ulnar artery and ulnar vein, or the radial artery and a venous perforator in the distal upper extremity to create the fistula. Endovascular fistula creation is an ideal option for patients who prefer to avoid or have contraindications to surgery. The WavelinQ and Ellipsys systems utilize different techniques, but both depend on the presence of a perforator vein to superficialize blood flow to the cephalic and basilic veins. The time needed for maturation for endovascular AVF is reported to be 90 (1–180) days using the WavelinQ system and 60 (1–164) days with the Ellipsys system. The reported average time for surgically created AVF maturation is 79 days (26). The rates of intervention were 27.7% for Ellipsys and 26.5% for WavelinQ. Current data suggest that surgically created AVFs frequently require additional intervention compared to EAVFs (26–28).

Studies on surgical vs. endovascular fistula creation are limited, but preliminary reports show that EAVFs may offer superior primary patency rates, lower intervention rates, and possibly lower overall costs than surgically created AVFs (26–28). Beathard et al. (29) reported a two-year cumulative review of proximal radial artery fistulas created by an endovascular approach with a 95% success rate. Success was considered a clinically functional AVF supporting two-needle dialysis according to the patient’s prescription. New generation devices have been developed since the time this manuscript was written (29). A smaller, 4F WavelinQ has been used in the EASE, EASE -2, and the EU postmarket clinical follow up study. A total of 116 patients underwent EAVF creation with a reported primary patency of 71.9%. The average time to maturity was 41 +/- 17 days, and the average time to successful cannulation was 68+/- 51 days. This new device allows percutaneous fistula creation between the radial artery and radial vein, or the ulnar artery and ulnar vein, making it a useful alternative to distal surgical AVF (30). Kitrou et al. (31) performed 30 consecutive endovascular AVF creation procedures with an excellent follow up of more than 500 days. The mean time to cannulation was 61.5 +/- 32.5 days. The patency rate was 96% at 1 year, and 82% at years 2 and 3. These data show very promising results for the endovascular creation of a distal AVF (31).

## Hemodialysis catheters

According to the USRDS (United States Renal Data System) data in 2018, 80.8% of patients initiated hemodialysis *via* a tunnelled central catheter (TCC). These statistics have not changed since 2009. This observation is striking but understandable. As Dr. Beathard stated in 1999 (32), hemodialysis catheters have many advantages. Because of their universal applicability, they can be easily inserted into multiple sites. They have a low cost of placement and replacement and do not require maturation. Catheters can be utilized in patients with acute kidney injury, in patients who present at stage 5 of chronic kidney disease, with a significantly reduced glomerular filtration rate that is approximately 15% or lower and need immediate initiation of hemodialysis, as well as for those who require long maturation of arteriovenous access, and in those with exhausted access sites (9).

Hemodialysis catheters are nontunnelled or tunnelled central catheters (TCCs). A nontunnelled central catheter is used for short-term emergent use. Nontunnelled catheters are made of polyurethane, which are somewhat stiff to facilitate entrance through the skin. This material becomes soft after placement when the catheter reaches body temperature (32, 33). The optimal position of the tip for nontunnelled catheters should be the superior vena cava. In the intensive care unit, a triple lumen nontunnelled catheter is suggested. A third, smaller medial lumen is helpful in critically ill patients. In chronic kidney disease patients who will need hemodialysis, it will help to preserve the integrity of veins for a potential AVF or AVG. A randomized trial has demonstrated similar infection rates for double- and triple-lumen nontunnelled catheters. Externally precurved catheters are preserved longer and have a significantly less bacteremia risk, as demonstrated by a historical analysis at the Vrije Universiteit Medical Center (Amsterdam, The Netherlands) (34). The general recommendation is to keep the indwelling catheter for less than two weeks (35). In areas of the world with limited resources, such as Latin America and Asia, temporary catheters are kept for as long as they remain infection-free and functioning, which sometimes could be up to 1 to 2 years (36).

**Figure 3:**
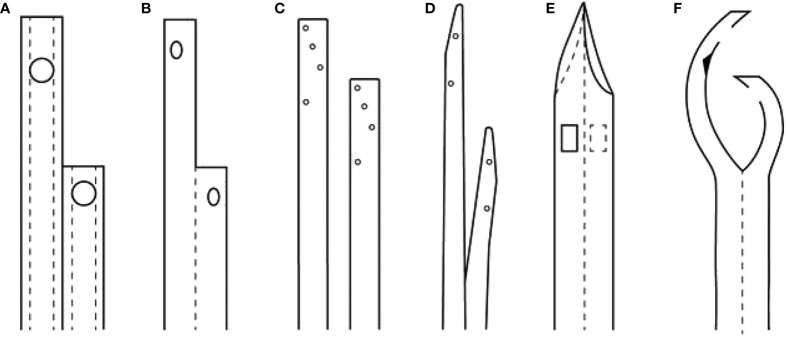
Comparison of the overall design of various CVC for maintenance hemodialysis, with axial cross-section of the catheters shows the locations of side holes and ports. **(A)** Quinton Perm Cath. **(B)** Mahurkar catheter, single body, DD design. **(C)** Canaud and Tesio twin catheters. **(D)** Ash split-tip catheter. **(E)** Symmetric-tip catheter by Tal. **(F)** SELF-CENTERING CATHETER (arrowhead indicates position of a self-healing hole to allow the catheter to be threaded over a single guide-wire or stylet) (34).

TCCs are more flexible and softer. Polycarbonate copolymers, such as Carbothane, are the most commonly employed materials for TCC coupling durability, softness, flexibility, and patient comfort. They have the advantages of polyurethane, but they can be made with thinner walls and greater strength. In addition, they are also resistant to iodine, peroxide, and alcohol. TCCs are available in sizes (15.5 or 16 French) that allow for blood flow rates higher than 300 mL/minute. Catheters have a variety of configurations and tip designs, including double D, coaxial, shotgun, step tip, symmetric, split-tip, and self-centered, among others (37, 38) (**Figure 3**).

Despite this wide variety of designs of TCCs, each with its theoretical advantages and disadvantages, the few available randomized trials have failed to show the superiority of one catheter over another, mainly when the endpoint is the long-term functional survival of the catheter. TCCs have significant problems and limitations. One of them is recirculation. In a group of 206 subjects, the straight tip, step tip, and split-tip TCC were studied for recirculation with standard and reverse connections. The recirculation was 0% for the straight tip, 39% for the split tip, and 16% for the step tip (37). Although not ideal, recirculation of the TCC is accepted in daily practice. Occasionally reversal of the lines -which increases recirculation-, is necessary in order to administer the dialysis treatment. Recirculation is associated with decreased clearances; however, many patients can maintain adequate KT/V in these circumstances. When reduced clearance and flow cause catheter dysfunction, the TCC should be replaced, as it will be discussed below.

As shown in **Figure 3F**, a new self-centering catheter designed with its central segments curved away from the vessel lumen to avoid/reduce the direct contact of the device with the vessel wall, thus preventing fibrin sheath formation has been developed. This catheter has demonstrated to have a high patency rate of approximately 90% after three months of use in different small trials. Although one of these trials was multicenter, none were prospectively controlled. Further data is needed to confirm these promising findings (39).

A major problem associated to TCCs is bloodstream infection (BSI). TCCs account for 70% of all access-related bacteremia’s in dialysis patients. In addition, patients with TCC have a 53% increased risk of all-cause mortality, a two and threefold higher risk for fatal and nonfatal infections, respectively, and a 68% higher risk of hospitalization than patients with AVF.

Multiple risk factors that interact with each other have been identified to play a role in catheter-related bloodstream infection (CRBSI). These include factors related to the host, such as impaired immunity, poor personal hygiene, occlusive dressing, nasal carriage of *Staphylococcus aureus*, older age, diabetes mellitus, recent hospitalization, and high cumulative doses of intravenous iron; factors related to the catheter, such as the site of insertion, prolonged use, history of bacteremia, colonization of the catheter tip and the cutaneous track with skin flora, catheter lumen contamination, hematogenous seeding from another infectious source, contamination of the lumen with the dialysate, and lack of aseptic precautions during catheter insertion; factors related to the pathogen including biofilm formation, resistance to antibiotics, bacterial virulence, and contiguous infection; and factors related to the hemodialysis procedure including contamination of the dialysate or equipment, inadequate treatment of water, and dialyzer reuse (35).

There are two ways organisms enter the bloodstream to cause CRBSI: through an extraluminal pathway and through an intraluminal pathway. The extraluminal pathway mainly occurs at the time of insertion of the catheter. The intraluminal pathway—the most common—involves the transfer of organisms by contact from the hands of individuals (usually healthcare workers) accessing the TCC, resulting in contamination of internal catheter surfaces (40, 41).

The best approach to prevent and treat CRBSIs has been evaluated in numerous studies. The current recommendations are as follows: 1) Blood cultures must be drawn before initiating antibiotic therapy. At present, the Infectious Disease Society of America (IDSA) allows the drawing of blood cultures from the dialysis lines to facilitate the capture of culture samples and to prevent interference with dialysis therapy. This simple step is necessary to establish a local identification of organisms and to streamline the antibiotic prescription when the results are available. Currently, the number of prescriptions of antibiotics at the dialysis units doubles the diagnosis of sepsis/bacteremia due to TCC in hemodialysis patients. This overuse of antibiotics is creating the emergence of multidrug-resistant organisms, particularly methicillin-resistant Staphylococcus aureus (MRSA) (20%) and, gram-negative resistant organisms (9%). 2) The initiation of therapy requires the use of broad-spectrum antibiotics according to the recommendations of the IDSA. 3) The catheter needs to be replaced over a wire or removed as soon as possible according to the clinical condition of the patient. 4) The presence of *Staphylococcus aureus* is a risk factor for developing serious metastatic complications. Approximately 3–44% of patients with Staph. aureus CRBSI can develop distant complications such as endocarditis, osteomyelitis, thrombophlebitis, septic arthritis, and spinal epidural abscess, among others. Therefore, treatment should include immediate removal of the catheter and administration of proper antibiotics delineated by the IDSA. 5) Exit site infection has been associated with a higher mortality rate and must be followed closely to evaluate the need for repositioning the exit site or changing the catheter site (40, 42, 43).

Different types of locking solutions have been designed to help prevent and control infection/thrombosis. This approach is helpful but costly and conveys the risk of creating multidrug-resistant organisms. The DOQI guidelines published in 2019 consider them a low-level recommendation. Currently, heparin is the best locking solution to prevent thrombosis. IDSA recommends using povidone-iodine antiseptic ointment or bacitracin/gramicidin/polymyxin B ointment at the hemodialysis catheter exit site after catheter insertion and at the end of each dialysis session only if this ointment does not interact with the material of the catheter. This benefit was validated in a meta-analysis performed in 2008 that showed a significant reduction in the rate of bacteremia, exit site infection (75 to 93%), and need for catheter removal/replacement. Bacitracin/gramicidin/polymyxin B ointment is not available in the U.S. Triple antibiotic ointment (bacitracin/neomycin/polymyxin B) is and may have a similar benefit but has not been adequately studied. New trials are necessary to evaluate this recommendation (40, 43–45).

Interestingly, a recent study reported an 83% reduction in the incidence of CRBSIs. This was achieved with stricter universal measures such as the use of the mask and the addition of consistent use of hydroalcoholic hand sanitizer due to the severe acute respiratory syndrome coronavirus 2 (SARS-CoV-2) pandemic. This study represents a small sample. However, the universal measures that have been proven to work were enforced more strictly. Further studies are needed to validate this observation (46). Other reports enforcing universal precautions have shown hemodialysis mortality and morbidity rates that were not associated with catheter complications (47).

Another publication that used data from the USRDS to examine the rates of antibiotic administration within dialysis facilities and the rates of hospital admission for CRBSIs and sepsis from March 2018 through November 2020 demonstrated that during the first 6 months of the pandemic, the rates of antibiotic administration were approximately 20% lower, and the rates of hospitalization for catheter-associated bloodstream infection were 24% lower than during corresponding periods in 2019. However, there were no significant changes in the rates of hospitalization for sepsis. These data correlate with the above-described observations that significant reductions in CRBSIs occurred during the pandemic and with strict enforcement of universal precautions. Therefore, it may be prudent to continue some mitigation of SARS-CoV-2 measures to prevent CRBSIs (48).

Catheter dysfunction: Chronic catheters are reported to develop a fibrous sheath immediately after insertion. These sheaths start at the entry site and continue along the entire catheter length, including the tip through which blood exchange is performed. This fibroelastic sheath will cause catheter dysfunction defined as 1) a decline in the blood flow rate of more than 10%, particularly if progressive, 2) arterial pressure (prepump) more negative than -250 mmHg or a venous pressure (postpump) higher than 250 mmHg, 3) a delivered Kt/V less than 1.2. Eventually, the dysfunction evolves into thrombosis or stenosis of the central veins. Thrombosis can be extrinsic or intrinsic. For intrinsic thrombosis, bedside measures such as reversal of ports, forceful saline flush, and injection of thrombolytics are recommended. If all this fails, the catheter usually needs to be replaced. Extrinsic thrombus could be localized in the central veins or the atrium, also known as catheter-related atrial thrombus (CRAT). Central vein thrombosis is managed with removal of the catheter and systemic anticoagulation for 3 months. For CRAT, if the thrombus is less than 2 cm, anticoagulation and catheter relocation to avoid further trauma in the thrombus area is recommended. For larger thrombi, anticoagulation directly through the catheter and systemic anticoagulation are the best choices (49, 50). No survival benefit has been observed from surgical embolectomy. However, if the patient has a contraindication to anticoagulation, if there is evidence of endocarditis, or if the thrombus is more than 6 cm, a surgical approach should be considered (49, 51, 52).

Recent studies have reported the prevalence of central vein stenosis (CVS) to be 10 to 28%. These findings are associated with long-term use of catheters, cardiac rhythm devices, and previous dialysis access or transplant history. Being older than 80 years of age seems to be a protective factor for the development of CVS according to these observations (53, 54). CVS is not associated with decreased survival per se. However, if the stenosis cannot be treated, the patient’s life can be compromised by the lack of an adequate access site for dialysis.

CVS is often asymptomatic in nondialysis patients but can result in edema of the ipsilateral extremity and breast when challenged by the increased flow from an arteriovenous fistula or graft. To understand CVS and improve the management, the Society of Interventional Radiology has classified central stenosis as thoracic central vein obstruction (TCVO). Four types have been described: type 1 involves the internal jugular and subclavian veins; type 2 affects the brachiocephalic vein on one side and the internal jugular veins; type 3 affects the bilateral brachiocephalic veins; and type 4 affects the superior vena cava. This classification aims to standardize the reporting and management of central lesions that predominantly affect dialysis patients (55).

TCVO should be treated with percutaneous transluminal balloon angioplasty alone or with a stent (bare-metal nitinol or stent-graft) (56). As mentioned earlier, hemodialysis vascular accesses with high-flow volumes benefit from flow reduction by banding the access inflow. This measure reduces the restenosis rate and helps to resolve the symptoms associated with a noncorrectable TCVO (57).

The treatment of TCVO could be complex, as more central vessels are involved and a higher level of expertise is required to avoid catastrophic complications. Interventionalists may need to utilize sharp or radiofrequency-assisted recanalization techniques with stenting to maintain the central vessels flow (56–58).

Recently, a new catheter system, “Surfacer Inside-out access,” has been used in patients with TCVO and enables right-sided placement of TCC across a range of obstruction types, including type 3 and 4 lesions. This procedure permits a right internal jugular approach going out of the vein through the stenosis to an exit site on the right side of the neck. It avoids going into the left internal jugular or femoral areas and preserves future access sites. It can be bridged to a Hemodialysis Reliable Outflow (HeRO) graft or arteriovenous graft to provide permanent access in dialysis patients. This technique requires careful evaluation with Doppler ultrasound and CT prior to the procedure to accurately locate the areas of stenosis and plan the intervention. Anesthesia or conscious sedation is used according to the severity of the lesion and general condition of the patient (59).

## Arteriovenous grafts

Arteriovenous grafts (AVGs) were initially introduced in 1972. First, a modified bovine carotid artery biologic graft (Artegraft, Johnson & Johnson) was used for vascular access in 8 hemodialysis patients and received some acceptance. However, in 1976, LD Baker Jr. (Phoenix, US) used expanded polytetrafluorethylene (ePTFE) in 72 hemodialysis patients. The majority were 8 mm AVGs. The rate of complications with ePTFE was no greater than those seen with the bovine heterograft. Additionally, its availability, ease of handling, biocompatibility, long-term stability, rapid use, and significant decrease in cost made the PTFE graft a first choice for surgeons (1, 60).

However, an evaluation by the USRDS on 5507 patients in December 1993 demonstrated a higher mortality risk for people with diabetes and nondiabetic patients with an AVG. AVGs were also associated with a higher risk of thrombosis and infection. Due to these findings, over the last several years, efforts have been directed to place an AVF in each patient. It is clear now that not all patients benefit from AVF placement due to poor maturation, thrombosis, and lack or exhausted venous vasculature. Therefore, the current approach is focused on “the right access for the right patient” (4, 61). From this perspective, some patients benefit from AVG placement to avoid the long-term use of TCCs. The decision to insert an AVG is determined after a complete history evaluation, physical examination, and vessel mapping to assess the arterial system and the draining veins. A minimum vein diameter of 4 mm is required for successful graft-vein anastomosis (62).

Prosthetic AVGs are either biological or synthetic. The biological grafts include denatured homologous vein allograft, cryopreserved saphenous vein, human umbilical vein, and sheep collagen grafts. Recently, bovine heterografts (Artegrafts) from carotid arteries with improved flexibility and patency were approved by the U.S. Food and Drug administration and possibly represent a safe alternative for patients with a history of multiple failed synthetic grafts. Seventeen Artegrafts were placed in 17 patients with a complex vascular access history in a small study. The 18-month primary patency was 73.3%, the primary assisted patency was 67%, and the secondary patency was 89%. Further studies have shown similar patency rates, with possible reduced infections and interventions (63).

At this time, the most frequently used synthetic graft is made of polytetrafluorethylene (PTFE), a fluorocarbon polymer. The stretch expanded (ePTFE) form is preferred based on a study by Tordoir et al. (64) and Akoh (65) who demonstrated, in a prospective comparison with standard PTFE (1995), a one-year cumulative patency rate of 59% versus 29% (p<0.01) for ePTFE. Through extensive research, new innovative forms of AVG have been developed, such as hybrid AVGs, heparin and drug eluting AVGs, hybrid AVG stents, anti-neointimal hyperplasia therapy, and the Intergraft Anastomotic Connector System (Phraxis, Inc., St. Paul, MN). Small trials were successful, but no superiority was confirmed.

An early cannulation arteriovenous graft (eAVG) is a novel graft with a trilayer design and a “self-healing” elastomeric membrane that allows rapid cannulation after insertion. eAVG is being used successfully in patients requiring emergent hemodialysis. A review of 19 studies utilizing eAVG’s Flixene (Marquet, USA), AVflo graft (Nicast Ltd, Israel), Acuseal graft (WL Gore, USA), and Vectra graft (Bard, USA) determined that early cannulation within 72 hours is possible. In addition, the 12-month follow-up demonstrated primary and secondary patency rates from 43 to 63% and 73 to 86%, respectively, which are acceptable. The use of eAVGs has also been linked to a savings of 11,000 dollars per year per patient due to fewer catheter complications and fewer secondary interventions. It is important to mention that these studies found infection rates similar to ePTFE grafts but higher than those of AVFs (65–69).

AVGs are associated with three significant complications, 1) infection, 2) stenosis/thrombosis, and 3) steal syndrome, referred now as hemodialysis access-induced distal ischemia (HAIDI).

1) AVG infection represents the most devastating form of infection in a dialysis patient. The most common organisms involved are *Staphylococcus aureus* 53%, methicillin-resistant S. aureus 17%, Coag-negative Staph sp. 10%, and Pseudomonas 8.5%. The most common presentations are bacteremia, sepsis, purulent drainage from the areas of cannulation, or exposed grafts. For proper treatment, total graft excision is necessary in 28-68% of patients. This requires insertion of a TCC until proper healing is achieved and is generally associated with a prolonged hospitalization for up to two weeks. Partial excision has been successfully attempted in approximately 40% of patients. When this approach is possible, it prevents the need for catheter use. However, this group of patients needs close follow-up since they require readmission and reintervention quite frequently. Interestingly, only 52% of patients obtained new access after one year of the infective episode, and sometimes, it was the nephrologist’s choice, possibly due to significant consequence on the patient’s overall health that this problem creates (70–72).

2) AVG stenosis: The primary patency rate for AVGs at one year is approximately 50%, and the failure rates increase by 0.8 to 1.0 events per patient per year. This poor outcome is due to the development of stenosis and thrombosis. It is well known that the development of intimal hyperplasia is the cause of stenosis. Stenosis occurs through a triad interaction between (1) biomaterial used (2); flow and blood properties such as shear rate and stress, flow rate oscillations and backflow, in addition to the interference of the uremic condition, coagulation, and inflammation; and (3) the geometrical shape of vessels and grafts, including the outer and inner diameter, length, and curvature with anticoagulation conditions. Intimal hyperplasia occurs in the juxta anastomotic segment of the outflow tract (73).

Until 2010, percutaneous transluminal angioplasty (PTA) was the best way to treat stenosis. In a total of 536 patients, Dr. Beathard demonstrated a success rate of 94% using angioplasty in fistulas and grafts. The average patency rate post angioplasty was estimated to be 60% at 6 months (74). In a 2010 study conducted by Haskal et al. (75), the Flair Endovascular Stent Graft (FSG) by Bard Peripheral Vascular was compared to angioplasty alone in functioning AVGs with significant venous anastomotic stenosis. At six months, the treatment area’s patency incidence was significantly greater in the FSG group than in the balloon angioplasty group. In addition, the patency of the access circuit was better in the FSG group. However, there was no statistically significant difference in the thrombosis rate (33% vs. 21%, p=0.10) (75).

In a second prospective, multicenter, randomized, concurrently-controlled post-approval study of the FLAIR endovascular stent graft (RENOVA), with the same FSG and with the follow-up period extended to 24 months after randomization, there was a significant improvement in the assisted primary patency at 12 and 24 months in the FSG cohort but no difference in the rate of thrombosis compared to the angioplasty group (44% vs. 36%, p=0.26) (76).

A prospective randomized comparison of balloon angioplasty versus the GORE VIABAHN stent graft device (VSG) (with CBAS Heparin Surface) (Gore & Associates) REVISE trial was another prospective, multicenter randomized controlled trial (RCT) conducted by Vesely et al. (77) The VSG was compared to PTA alone in both functioning and thrombosed AVG with venous anastomotic stenosis. At 6 months, the target lesion primary patency was significantly better for the VSG than PTA alone. VSG delayed the recurrence of stenosis compared to PTA, despite whether the AVG was open or thrombosed at the time of randomization. Further analysis of the REVISE trial results supports the primary use of stent grafts for venous anastomotic AVG stenosis, particularly in AVG thrombosis, where stent grafts provide overall cost savings by decreasing the number of interventions. The PTA outcomes at six months were noted to be lower than those reported earlier, and the reason is unknown. It seems to correlate with the use of high-pressure balloons (76–79).

Once a stent is placed in the access, it would seem difficult for the stenosis to recur, but restenosis is common. For bare-metal stents (BMSs) the restenosis usually occurs within the stent (in-stent), whereas stenosis typically occurs at the edges of the stent graft (in-segment). Falk et al. (80) conducted a prospective, randomized study of an expanded polytetrafluoroethylene stent graft versus balloon angioplasty for in-stent restenosis in AVGs and AVFs (RESCUE), which compared angioplasty alone to angioplasty and stent-graft placement as treatment for in-stent stenosis following BMS placement in the outflow tract of AVGs and AVFs with a follow-up of two years. The results showed a significant advantage of stent grafts in decreasing restenosis compared to angioplasty alone (15.6% vs. 2.2%, p<0.001, TAPP). However, at two years, the access circuit patency was almost zero between the two groups (0.9% vs. 0.8%). Overall, these studies demonstrate that stent placement maintains the primary site patency significantly better than PTA alone; however, there is no significant access survival benefit (76, 80).

3) AVG Thrombosis: AVG thrombosis occurs approximately <0.5–2.0 times per year and AVF thrombosis occurs 0.1 to 0.5 times per year (81). This signifies a true emergency for the dialysis patient, since it can affect the overall health of patients undergoing dialysis. It can cause electrolyte abnormalities such as life threatening hyperkalemia, hypervolemia and complications related to the use of catheters. It is mandatory that a procedure to restore flow be performed within 48 hours of the event to rescue the access. The ultimate goals are to prevent the use of a tunneled HD catheter and hospitalization. Thrombectomy is also attempted in patients with AVGs that thrombose a few days after placement. Usually after two to three weeks post surgery it is possible to reestablish flow and use these accesses. For functional av fistulas, a mechanical thrombectomy with balloon angioplasty is a minimally invasive and effective procedure for the treatment of a thrombosed native arteriovenous fistula (82).

Eighty to ninety percent of AVG thrombosis cases are due to stenosis of the venous anastomosis. Different techniques are used to reestablish flow, including surgical and endovascular techniques. Surgical thrombectomy is the classic approach *via* new anastomosis or patch angioplasty. Another surgical technique is the manual removal of the clot followed by angioplasty of the different lesions.

Endovascular thrombectomy can be performed using pharmacological treatments, including fibrinolytic agents such as tPA or Urokinase, and then waiting. Another method is the spray-pulse, in which the fibrinolytic agent is injected by pulses according to the clot burden. Other methods include thromboaspiration of the clot or pharmacomechanical thrombectomy, in which an injection of a thrombolytic agent induces thrombolysis and then an angioplasty is performed to treat the stenosis and mobilize the clot to the central circulation. Another technique is the mechanical thrombectomy. In this case, the clot is extracted through a device such as the “Arrow-Trerotola” and then a fibrinolytic agent or a balloon is used to mobilize the clot and treat the stenosis.

A recent meta-analysis of the different techniques for thrombectomy showed that the outcomes of endovascular and surgical interventions for a thrombosed vascular access are comparable, particularly for thrombosed prosthetic grafts. Endovascular treatment is less invasive and allows preservation of the site. One negative aspect of the endovascular procedure is that it could require a higher number of interventions in some patients. Possible complications of the endovascular technique are pulmonary embolism, arterial embolus, graft rupture, hematoma or vein dissection and, if using thrombolytics, prolonged bleeding (83). In the opinion of these authors pharmaco-mechanical thrombectomy is a practical, inexpensive and reproducible way to successfully treat AVG thrombosis.

Different studies on pharmacological therapy for fistula maturation and stenosis prevention have not shown significant benefit. Various trials showed that anticoagulation therapy, including the use of heparin or oral anticoagulants, had higher risks of bleeding complications and did not prevent access failure. Likewise antiplatelet aggregation with agents such as dipyridamole and clopidogrel increased the risk of bleeding events and did not prevent AVG thrombosis (84). Omega 3 polyunsaturated fatty acids (PUFAs) have anti-inflammatory, antiproliferative, anti-platelet aggregation activity and vasodilatory effects, and have been used in different clinical trials to assess their benefits on the prevention of stenosis. Viecelli et al. (85) performed a meta-analysis of five RCTs. They concluded that omega-3 PUFA supplementation started at the time of arteriovenous access surgery may prevent primary patency loss within 12 months but may have little or no effect on access interventions, access failure or access abandonment, and treatment harms are uncertain (85). Balloon-assisted maturation is performed by a repeated, long segment angioplasty of the peri-anastomotic venous segment along the venous outflow, thus dilating the vein in staged sessions. This procedure, thought to cause more rapid outward remodeling of the venous limb and allowing for quicker maturation, has been relatively successful in several centers (86).

A measure that is considered important to prevent thrombosis is access surveillance. According to the KDOQI guidelines, a flow rate less than 600 ml/min or a decrease of 25% over a period fewer than four months is indicative of a significant stenotic lesion, and a fistulogram is required. Despite surveillance and timely interventions, some grafts do develop clots. A significant percentage of graft thrombosis that does occur with access flow surveillance occurs in AVGs with preserved flows. In a single center observational study from 2006 to 2014 by Magbri et al. (87), it was demonstrated that the total number of thrombectomies per year decreased from 94 to 42 by using a surveillance program. The number of angioplasties doubled. It is unclear whether this methodology would help to preserve the long-term patency. This topic has been controversial and discussed in many publications. Some elegant studies have reported against surveillance (88, 89). Nevertheless, it is valid to acknowledge that even though surveillance programs may not prolong long-term patency, it is true that performing an angioplasty is much easier than a thrombectomy, costs less, and does not disrupt the patient’s care (90). Interventions are helpful to facilitate cannulation and support adequate dialysis on a regular basis. After much discussion, it is agreed that a thorough physical examination with a clinical surveillance program by experienced health care professionals should be the recommended procedure to evaluate the functional status of the vascular access and will help to prevent thrombosis (89, 90).

4) Hemodialysis access-induced distal ischemia (HAIDI), a challenging complication of AVGs and AVFs, is symptomatic extremity ischemia caused by the diversion of arterial flow through the access site. An incidence between 1 and 8% is reported; however, this number may not be accurate since there is a gamut of symptomatology that qualifies for the syndrome **Table 1**.

**Table 1:** Clinical classification of HAIDI.

Stage	Signs and symptoms	Management
Stage 1	No clear symptoms.only signs	Conservative management
	Nail beds slightly cyanotic and/or pale. mild coldness of skin of hand, decreased pulse at wrist	Close observation
Stage 2a	Nail beds cyanotic or pale. coldness of skin of hand, dec.reased pulse at wrist. tolerable pain, cramps, paraesthesia, numbness during dialysis or with exercise of hand	As above^a^ plus
		Access blood flow measurement
		Low-surgical referral
		High-consider flow reduction
		Angiography
		Treat arterial stenosis
Stage 2b	Nail beds cyanotic or pale, coldness of skin of hand. decreased pulse at wrist intolerable pain, cramps.paraesthesia, numbness during dialysis or with exercise of hand	Intervention
		Access blood flow measurement
		Low-surgical referral
		High-consider flow reduction
		Angiography
		Treat arterial stenosis
		As above
Stage 3	As above plus	Early treatment is indicated
	Rest pain or motor dysfunction of fingers or hand	As above
Stage 4a	Tissue loss-ulceration.necrosis	Emergent intervention required
	Motor and/or sensory loss	As above
Stage 4b	Extensive. extensive tissue loss	Urgent intervention required
		Consider closure of access
		Amputation may be necessary

HAIDI, hemodialysis access-induced distal ischemia Beathard et al. (29).

Three etiological factors have been identified as causing HAIDI: arterial stenosis, high fistula flow and lack of vascular adaptation or collateral flow. It can be immediate, in which case ligation of the access may be necessary, or it could be late onset, as the fistula flow increases over time. A rare immediate complication is ischemic monomelic neuropathy (IMN), which has a similar pathogenicity. IMN is characterized by acute pain, paresthesia, and weakness immediately following the access creation, which is usually in the arm, and it is also an indication for access ligation to prevent permanent sensory and motor damage (29, 91).

There is no reliable method to predict HAIDI, and the different tests available, including digital pressure to assess the vasculature, are not entirely reliable. HAIDI treatment should be directed to improve the distal flow and rescue the access when possible. This task can be challenging and often depends on the surgeon’s approach and experience managing the vascular access.

A detailed review of the different methods to treat this problem is beyond the scope of this report. However, it is important to mention that two methods are preferred: 1) banding, which reduces the flow to the access. It is simple, provides immediate improvement of the symptoms, and is performed with local anesthesia, and 2) surgical procedures in which distal revascularization interval ligation (DRIL), although complex to perform, addresses the cause of ischemia and rescues the access. Proximalization of the inflow is also recommended with graft placement to prevent HAIDI (29, 65).

5) New Directions New methods of optimization of AVGs include tissue-engineered grafts from synthetic materials or biopolymers. Different trials in small and large animals have demonstrated that using a biocompatible, biodegradable “scaffold” made as a vascular structure could show excellent biocompatibility and mechanical properties over two years. After implantation, the autologous host cells repopulate the scaffold wall, producing a new conduit while the polymer degrades (73). A human acellular vessel (HAV) for dialysis has also been used. Recently a Five year outcome in patients with ESKD who received the bioengineered human acellular vessel for dialysis access in a Phase 2 clinical trial was reported. Eleven patients completed at month 60. One patient maintained primary patency, and 10 maintained secondary patency. Secondary patency was estimated at 58.2% (95% confidence interval 39.2–73.1) at five years, after censoring for deaths (*n* = 8) and withdrawals (*n* = 1) and no infection was reported. This type of graft may provide a durable and functional access for ESRD patients (92). Autologous biotube, a graft that is grown inside the host by implanting a foreign body precursor in the shape of a rod, is being explored. Studies have been performed on animals and have been successful. Cost, scale, manufacturing, 3D printing, durability, biocompatibility, and thrombogenicity are important questions that are being investigated with long-term clinical studies in the search for the “ideal vascular access” (73).

## Conclusion

Providing adequate vascular access for hemodialysis patients poses a significant challenge due to the difficulty of finding the ideal access for each patient. Based on the premise that AVF is the best known access and the fistula first initiative, AVF use has greatly increased. This is beneficial for many patients. However, patients whose fistulas do not mature and require endovascular interventions to reach the functional state need to start hemodialysis with a TCC. This fact is associated with high morbidity and mortality. To address this issue, an accurate and standardized indicator to predict the chances of fistula maturation in any given patient is needed. Using this indicator, the decision to place an access needs to be based on the highest standard of care and the knowledge that fistulas are the best access that we can provide for a patient. In patients who would be candidates for a fistula, the temporary use of a TCC should not be automatically avoided in favor of an AVG. Access planning *via* vein mapping and vein preservation needs to be the number one priority in patients who initiate hemodialysis with a catheter. The access prescription should be as important as the dialysis prescription.

Multiple efforts are focused on making AVGs more biocompatible, less prone to infection, and more durable. Reports on bovine grafts are positive, but more studies are needed. EAVFs are being created more frequently and appear to be less prone to complications (26, 63, 73).

All the vascular advancements discussed above will not be effective if dialysis personnel and patients are not educated to properly care for vascular accesses. Patients must learn and understand that “their access is their lifeline.” Patients and dialysis personnel must work together initially planning for permanent access when needed, observing good hygiene before and after the dialysis session, learning about the different types of accesses, and proper cannulation techniques to improve the longevity of the access. NTDS initiative to encourage prevention, education, and a culture where all parties feel safe reporting incidents is also very important.

Last, the SARS-CoV-2 pandemic has left us with a valuable lesson. The marked reduction in infections worldwide in dialysis units demonstrates that by enforcing universal precautions, it is possible to prevent and reduce infections in a low-cost manner and improve the morbidity/mortality of the dialysis population.

New technologies and research are important in the search for an ideal access that will allow ESRD patients to be healthier and reduce Medicare expenses. However, we cannot focus all our efforts on costly devices and interventions. Simple and low-cost solutions such as education and prevention will play a crucial role in improving the vascular access panorama.

## Author contributions

NN reviewed the manuscript. Both authors worked closely on the different topics.

## Acknowledgments

The authors are very grateful to Renée Grange for her tireless and thorough editorial work on this manuscript.

## Conflict of interest

The authors declare that the research was conducted in the absence of any commercial or financial relationships that could be construed as a potential conflict of interest.

## Publisher’s note

All claims expressed in this article are solely those of the authors and do not necessarily represent those of their affiliated organizations, or those of the publisher, the editors and the reviewers. Any product that may be evaluated in this article, or claim that may be made by its manufacturer, is not guaranteed or endorsed by the publisher.

## Abbreviations

AVF Arteriovenous Fistula; AVG Arteriovenous graft; BMS Dare Metal Stents; BSI Blood Stream infections; CRBI Catheter Related Bloodstream Infection; CDC Centers for Disease Control and Prevention; CMS Centers for Medicare & Medicaid Services; CRAT Catheter Related Atrial Thrombus; CT Computed Tommography; CVS Central Vein Stenosis; DCB Drug Coated balloon; EAVF Endovascular Arteriovenous Fistula; eAVG Early cannulation Arteriovenous Graft; ESKD End Stage Kidney Disease; FSG Flair Endovascular Graft; FFI Fistula First Initiative; FIR Far Infrared Therapy; HAIDI Hemodialysis Access Induced Distal Ischemia; HeRO Hemodialysis Reliable Outflow; IDSA Infectious Disease Society of America; K-DOQI Kidney Disease Dialysis Outcomes and Quality Initiatives; KT/V Dialysis Clearance index; MCP-1 Monocyte Chemoattractant Protein 1; MRSA Methicillin-resistant Staphylococcus aureus; NHSN National Healthcare Safety Network; NTDS Nephrologists Transforming Dialysis Safety; Omega-3 PUFAs Omega -3 Polyunsaturated Fatty Acids; PTA Percutaneous Transluminal Angioplasty; PTFE Polytetrafluoroethylene; ePTFE Expanded Polytetrafluoroethylene; RCT Randomized controlled trial; RENOVA Post-Approval Study for the FLAIR Endovascular Stent Graft; REVISE Vascular Access Revision with Viabahn Endoprosthesis vs. Percutaneous Transluminal Angioplasty; SAR CoV-2 severe acute respiratory syndrome coronavirus 2; TCC Tunnelled Central Catheter; TCVO Thoracic Central Vein Obstruction; TNF-Alpha Tumor Necrosis Factor; t-PA Tissue Plasminogen Activator; USRDS United States Renal Data Systems; VSG Viabahn Stent Graft

## Glossary

**Table:**

AVF	Arteriovenous Fistula
AVG	Arteriovenous graft
BMS	Dare Metal Stents
BSI	Blood Stream infections
CRBI	Catheter Related Bloodstream Infection
CDC	Centers for Disease Control and Prevention
CMS	Centers for Medicare & Medicaid Services
CRAT	Catheter Related Atrial Thrombus
CT	Computed Tommography
CVS	Central Vein Stenosis
DCB	Drug Coated balloon
EAVF	Endovascular Arteriovenous Fistula
eAVG	Early cannulation Arteriovenous Graft
ESKD	End Stage Kidney Disease
FSG	Flair Endovascular Graft
FFI	Fistula First Initiative
FIR	Far Infrared Therapy
HAIDI	Hemodialysis Access Induced Distal Ischemia
HeRO	Hemodialysis Reliable Outflow
IDSA	Infectious Disease Society of America
K-DOQI	Kidney Disease Dialysis Outcomes and Quality Initiatives
KT/V	Dialysis Clearance index
MCP-1	Monocyte Chemoattractant Protein 1
MRSA	Methicillin-resistant Staphylococcus aureus
NHSN	National Healthcare Safety Network
NTDS	Nephrologists Transforming Dialysis Safety
Omega-3 PUFAs	Omega -3 Polyunsaturated Fatty Acids
PTA	Percutaneous Transluminal Angioplasty
PTFE	Polytetrafluoroethylene
ePTFE	Expanded Polytetrafluoroethylene
RCT	Randomized controlled trial
RENOVA	Post-Approval Study for the FLAIR Endovascular Stent Graft
REVISE Vascular Access Revision with Viabahn Endoprosthesis vs.	Percutaneous Transluminal Angioplasty
SAR CoV-2	severe acute respiratory syndrome coronavirus 2
TCC	Tunnelled Central Catheter
TCVO	Thoracic Central Vein Obstruction
TNF-Alpha	Tumor Necrosis Factor
t-PA	Tissue Plasminogen Activator
USRDS	United States Renal Data Systems
VSG	Viabahn Stent Graft
